# Editorial: Human-Centered Artificial Intelligence in Industry 5.0

**DOI:** 10.3389/frai.2024.1429186

**Published:** 2024-06-10

**Authors:** Gregoris Mentzas, Karl Hribernik, Johan Stahre, David Romero, John Soldatos

**Affiliations:** ^1^School of Electrical and Computer Engineering, National Technical University of Athens, Athens, Greece; ^2^BIBA - Bremer Institut für Produktion und Logistik GmbH at the University of Bremen, Bremen, Germany; ^3^Chalmers University of Technology, Göteborg, Vastra Gotaland County, Sweden; ^4^Tecnológico de Monterrey, Mexico City, Mexico; ^5^INTRASOFT International, Luxembourg, Luxembourg

**Keywords:** Artificial Intelligence, human-centered, manufacturing, Industry 5.0, machine learning, data analytics

## Introduction

Over the past years, Industry 4.0 has evolved into a widely recognized concept worldwide. Numerous nations have launched analogous strategic endeavors, dedicating significant research to advancing and integrating multiple Industry 4.0 technologies. As the 10-year milestone of Industry 4.0′s inception approached, the European Commission unveiled the concept of “Industry 5.0” (European Commission, 2021).

Industry 5.0 places the worker at the center of the production process and uses new technologies to provide prosperity beyond jobs and growth, while respecting the production limits of the planet. It complements the Industry 4.0 approach by putting research and innovation at the service of the transition to a human-centric, sustainable, and resilient industry. Xu et al. (2021), Leng et al. (2022), and Ivanov (2023) give overviews of this evolution, while Akundi et al. (2022) analyze the state of Industry 5.0 and outline research trends.

The use of Artificial Intelligence (AI) in Industry 4.0 has provided solutions that leverage data available from smart sensors, devices, and machines to enable the generation of actionable intelligence and help increase manufacturing efficiency (Peres et al., 2020; Jan et al., 2023). However, this evolution in the use of AI has not been accompanied by similar emphasis and progress on fundamental aspects of human-centered processes and systems. Human-centered AI (HCAI) focuses on creating systems designed and developed by augmenting human intelligence with machine intelligence (Shneiderman, 2020a,b).

Given that Industry 5.0 puts emphasis on the human factor and considers it at the center of production, it is only natural that HCAI is called on to support the migration to Industry 5.0 since humans will have to collaborate with digital solutions such as AI systems, robots, etc. This trend extends the research efforts toward the “Operator 4.0” and their interaction with AI and robotic systems (Bousdekis et al., 2020; Romero et al., 2020).

The focus of this research is on advancements in HCAI systems for Industry 5.0. Such systems view AI as a critical component for augmenting human work, extending human capabilities within an industrial environment. Thus, they are key enablers for the “Operator 5.0” concept (Romero and Stahre, 2021; Gladysz et al., 2023).

The aim of the Research Topic is to explore methods, tools, and cases in which AI systems and humans work as teams within industrial settings in order, to jointly solve problems and achieve goals that were unreachable by either humans or machines alone (Alves et al., 2023; Pizoń and Gola, 2023).

In the call for submissions for this Research Topic we listed the following specific themes:

- Design issues for multimodal human-AI interactions in the industrial environment.
- Digital intelligent assistants, softbots, and chatbots for production management.
- Artificial Intelligence for the operator in the Industry 5.0 environments.
- Cognitive computing and HCAI engineering.
- Explainable and transparent AI in smart manufacturing.
- Usability and user experience of HCAI systems.
- Methods and tools to manage and monitor industrial HCAI systems.
- Applications of HCAI systems in smart manufacturing.
- Human-centered security and privacy issues in AI deployments.
- Evaluation and performance metrics of HCAI case studies.
- Cases and lessons learned from HCAI industrial experiments or large-scale rollouts.

Five papers were accepted, which will be summarized next. They address a wide portfolio of topics ranging from technological approaches, for example, on the use of multi-agent systems for integrating human characteristics into production systems, explaining AI algorithms to human users, up to the impact of AI on employment patterns in the manufacturing industry.

In “*The MAS4AI framework for human-centered agile and smart manufacturing*,” Sidorenko et al. argue that the software agent technology offers a promising avenue for developing interoperable software applications in modern production systems. They propose Multi-Agent Systems (MASs) as viable for implementing Industry 4.0 components. However, because recent approaches to MASs in manufacturing systems face limitations, such as centralized coordinating agents and low automation levels, the paper focuses on enhancing MAS-based approaches for reconfigurable manufacturing systems while considering Industry 4.0 concepts. The authors address questions like improving collaboration among heterogeneous production assets, integrating human characteristics into production systems, and achieving effective task sharing between humans and machines. The paper presents a multi-agent framework that extends existing approaches by introducing the concept of Human Digital Holon for enhancing human-system integration. Based on the RAMI 4.0 model, the framework utilizes Asset Administration Shell (AAS) for digital representation, promoting interoperability. Human-system integration is achieved by modeling various human aspects as AAS submodels and augmenting human behavior with a digital holon. The framework, conceptualized in a prototype, is currently undergoing testing in industrial use cases, with two scenarios demonstrating human integration into shared human-machine tasks.

In “*Explainability as the key ingredient for AI adoption in industry 5.0 settings*,” Agostinho et al. focus on Explainable AI (XAI) models that offer insights into AI system decision-making. They argue that in dynamic environments like manufacturing, XAI often struggles with complex problems due to numerous parameters involved. They describe the XMANAI project that addresses this challenge by balancing transparency and accuracy, focusing on customizable XAI solutions for manufacturing. The paper outlines the approach used to develop the XMANAI platform, emphasizing security, interoperability, transparency, and asset lifecycle management. The platform spans three dimensions: Data, Services, and AI models, ensuring secure handling of datasets, AI algorithms, and models while addressing manufacturing use cases. XMANAI aims to provide a secure environment for data scientists and business users to create interpretable AI solutions, bridging the gap between black box models and domain knowledge. The platform enables collaborative construction of transparent AI models, facilitating informed decision-making in manufacturing. The paper also introduces an evaluation framework, utilizing methodologies like the extended 6P methodology and Fuzzy Cognitive Maps, assesses the business value and impact of XAI in manufacturing.

In “*Knowledge sharing in manufacturing using LLM-powered tools*,” Freire et al. address a challenge that persists in effectively managing and utilizing manufacturing knowledge, due to processing difficulties and unstructured technical information. Large Language Models (LLMs), like GPT-4, offer promise by interpreting vast text-based datasets and aiding in knowledge capture. However, applying LLMs in manufacturing faces unique challenges, including customization needs and socio-technical risks. To address this, the paper describes an LLM-powered tool that was developed to answer operator queries and facilitate issue analysis in factories. The tool demonstrates the feasibility of using LLMs to enhance knowledge management in manufacturing settings and was evaluated through a user study. Additionally, the paper describes specific benchmarks to assess LLM performance in manufacturing contexts, focusing on factual and complete answers to operator queries. This work highlights the potential of LLMs to improve knowledge utilization in manufacturing while addressing the challenges unique to this domain.

In “*tachAId—an interactive tool supporting the design of human-centered AI solutions*,” Bauroth et al. argue that although Human-Centered AI (HCAI) aims to create collaborative AI systems that enhance human capabilities, many AI solutions overlook potential human impacts, leading to suboptimal performance or even harm. In order to address such issues the paper argues that there is a need for designing AI solutions specifically adjusted to human needs in a work environment. The paper addresses this need by proposing tachAId, an interactive tool, designed to aid company stakeholders and AI developers to design human-centered AI solutions. tachAId guides users along the phases of AI development, points at potential challenges at the points of contact between humans and AI, and maps these challenges to technical measures and tools, for example, in the form of algorithms or libraries, that can be used to satisfy diverse requirements toward HCAI.

Finally, in ““*Machine replacement” or “job creation”: How does artificial intelligence impact employment patterns in China's Manufacturing Industry?*,” Huo et al. argue that it is essential to examine whether AI integration leads to job displacement or creation, given the dynamic international environment and non-systemic shocks. This study analyzes the impact of AI on employment patterns in the manufacturing industry using task models and empirical data from China's manufacturing sector from 2011 to 2020. The findings indicate a positive U-shaped relationship between AI development and total employment, with short-term effects driven by substitution and long-term effects by creation. Low-skilled labor is more susceptible to replacement, while industries like finance and hospitality show less impact from manufacturing industry spillovers. Moreover, there is evidence of improved employment quality, narrowing the urban-rural income gap. To address AI's impact on employment patterns, strategies such as boosting AI development, expanding job opportunities, enhancing skill training, and promoting regional integration are recommended. These measures aim to ensure that technological progress benefits all sectors of society.

## Conclusions

As we traverse through the evolution of industry, from the mechanization of Industry 1.0 to the digitalization of Industry 4.0, one overarching theme becomes increasingly clear—the symbiotic relationship between human ingenuity and technological advancement. Now, as we stand on the cusp of Industry 5.0, characterized by the integration of advanced technologies such as Artificial Intelligence (AI), the emphasis on human-centered approaches is more crucial than ever before.

At the heart of Industry 5.0 lies the recognition that while automation and AI can revolutionize efficiency and productivity, they must also preserve and enhance the essence of human contribution. Human-Centered Artificial Intelligence (HCAI) therefore, assumes a pivotal role in shaping this industrial era. Human-centered AI prioritizes the augmentation, rather than the replacement, of human capabilities. Rather than viewing AI as a substitute for human labor, Industry 5.0 envisions it as a tool to empower individuals to perform tasks more efficiently and effectively. By leveraging AI technologies such as machine learning and natural language processing, workers can offload mundane and repetitive tasks, allowing them to focus on more creative and strategic endeavors. This augmentation amplifies human potential, leading to greater innovation and problem-solving within organizations.

Furthermore, human-centered AI prioritizes transparency and accountability in decision-making processes. As AI systems become increasingly autonomous, it becomes imperative to demystify their inner workings and ensure that they align with high ethical and moral standards within trustworthy systems (Díaz-Rodríguez et al., 2023; Li et al., 2023; Mentzas et al., 2024). Industry 5.0 promotes the development of AI algorithms that are explainable and interpretable, allowing humans to comprehend and scrutinize their outputs and thereby lead to trustworthy AI within industrial settings. By integrating ethical considerations into the design and deployment of AI systems, Industry 5.0 safeguards against unintended consequences and promotes trust between humans and machines (Vyhmeister and Castane, 2024).

The papers in this Research Topic demonstrated that Human-Centered Artificial Intelligence (HCAI) lies at the core of Industry 5.0, driving a paradigm shift that emphasizes the synergistic relationship between humans and technology. By augmenting human capabilities and ensuring transparency as well as high ethical standards, HCAI ensures a framework for industry 5.0 that combines competitiveness and sustainability, allowing industry to realize its potential as one of the pillars of the digital transformation.

## Author contributions

GM: Writing – original draft, Writing – review & editing. KH: Writing – review & editing. JSt: Writing – review & editing. DR: Writing – review & editing. JSo: Writing – review & editing.
